# A Risk-Stratified, Volume-Based Plus Enteral Nutrition Protocol with Semi-Elemental Formula in Critically Ill Surgical Patients: A Before-and-After Implementation Study

**DOI:** 10.3390/medicina62081591

**Published:** 2026-08-18

**Authors:** Pawit Aue-apinya, Gelan Miao, Kaweesak Chittawatanarat, Srisuluk Kacha, Natsuda Phothikun, Atirut Supphapipat

**Affiliations:** 1 Division of Surgical Critical Care, Nutrition and Metabolism, Department of Surgery, Faculty of Medicine, Chiang Mai University, Chiang Mai 50200, Thailand; pawit.a@cmu.ac.th (P.A.-a.); gelan_miao@cmu.ac.th (G.M.); srisuluk.ka@cmu.ac.th (S.K.); natsuda.dokkaew@cmu.ac.th (N.P.); atirut_s@hotmail.com (A.S.); 2 Clinical Surgical Research Center, Department of Surgery, Faculty of Medicine, Chiang Mai University, Chiang Mai 50200, Thailand; 3 Department of Anesthesiology, Faculty of Medicine, Chiang Mai University, Chiang Mai 50200, Thailand

**Keywords:** enteral nutrition, volume-based feeding, refeeding syndrome, critical care, nutritional protocol, caloric deficit

## Abstract

*Background and Objectives*: Critically ill surgical patients frequently fail to achieve prescribed enteral nutrition targets because of feeding interruptions and variability in conventional physician-directed feeding practices. Although volume-based feeding (VBF) has been proposed to improve nutritional delivery, concerns remain regarding refeeding syndrome and gastrointestinal intolerance. The aim of this study was to evaluate whether a Volume-Based Plus (VBF+) protocol integrating volume-targeted feeding with mandatory refeeding risk stratification and a standardized semi-elemental enteral formula improves nutritional delivery without increasing gastrointestinal or metabolic complications. *Materials and Methods*: We conducted an ambispective before-and-after implementation study (retrospective control phase, prospective intervention phase) in the surgical intensive care unit of a tertiary care center between January 2023 and March 2026. Ninety-six patients requiring enteral nutrition for ≥3 days were enrolled (48 control and 48 intervention). Both groups underwent the same refeeding risk stratification to guide feeding strategy, while the intervention group additionally received the standardized VBF+ protocol with risk-stratified caloric advancement and volume-based compensation for feeding interruptions, and a standardized semi-elemental enteral formula. The primary outcomes were median daily caloric delivery (% of target) and mean daily protein delivery (g/kg/day). Multivariable regression analyses adjusted for Nutritional Assessment Form (NAF), APACHE II score, and Charlson Comorbidity Index (CCI) were performed. *Results*: The VBF+ protocol was independently associated with higher caloric delivery (β = 31.2 percentage points, 95% CI 17.6 to 44.8; *p* < 0.001) and higher protein delivery (β = 0.168 g/kg/day, 95% CI 0.040–0.297; *p* = 0.011). Longitudinal analysis showed a significantly faster increase in protein delivery in the intervention group (*p* < 0.001), while the group-by-time interaction for caloric delivery did not reach statistical significance (*p* = 0.073). Rates of gastrointestinal intolerance and metabolic complications were comparable between groups; as VBF+ bundled the compensatory algorithm with a semi-elemental formula, this gastrointestinal benefit is hypothesis-generating. *Conclusions*: In this before-and-after study, the VBF+ bundle protocol was associated with improved caloric and protein delivery and reduced gastrointestinal intolerance; because the algorithm and formula were not evaluated independently, the gastrointestinal benefit remains hypothesis-generating and requires confirmation in randomized trials.

## 1. Introduction

Providing adequate enteral nutrition (EN) to critically ill patients remains a significant clinical challenge. Observational data consistently demonstrate that ICU patients receive only 50–60% of their prescribed caloric requirements [1], resulting in a cumulative caloric deficit that is independently associated with adverse clinical outcomes including infection rates, prolonged mechanical ventilation, extended ICU length of stay, and higher mortality [2].

The prevailing standard of care uses a rate-based feeding (RBF) strategy, in which a fixed hourly infusion rate is calculated to meet the patient’s 24-h caloric prescription. This model is inherently vulnerable in the dynamic ICU environment: feeding is frequently interrupted for procedures, airway management, or intolerance [3,4], and when resumed, the infusion rate is restarted at the same fixed rate without compensating for the lost volume. This systematic failure to recover interrupted feeding time ensures that patients consistently receive less nutrition than prescribed [5].

Volume-based feeding (VBF) addresses this limitation by shifting the clinical goal from maintaining a fixed hourly rate to achieving a prescribed total 24-h volume. The key innovation is empowering nursing staff to adjust the infusion rate during available feeding windows to compensate for unavoidable interruptions. A randomized controlled trial by McClave et al. demonstrated that patients receiving VBF achieved 92.9% of their caloric goal compared with 80.9% in the RBF group (*p* = 0.01), with significantly smaller caloric deficits [5]. Subsequent studies in surgical and trauma populations have replicated these findings [6].

However, widespread implementation of VBF has been limited by safety concerns, particularly regarding refeeding syndrome (RFS). RFS is a potentially life-threatening metabolic complication characterized by rapid electrolyte shifts—predominantly hypophosphatemia, hypomagnesemia, and hypokalemia—precipitated by re-initiation of nutrition in malnourished or chronically undernourished patients [7]. Existing VBF protocols do not incorporate systematic risk stratification for RFS, which limits their safe application in high-risk populations [8]. Furthermore, not all ICU patients are candidates for immediate full-volume feeding; hemodynamically unstable patients and those with specific surgical contraindications benefit from trophic feeding—minimal enteral delivery designed to maintain gut integrity without imposing metabolic demands [9].

To address these gaps, we developed the Volume-Based Plus (VBF+) protocol, which integrates volume-based delivery with mandatory refeeding risk stratification and explicit trophic feeding criteria, and a standardized semi-elemental enteral formula. The primary objective of this study was to evaluate whether implementation of the VBF+ protocol results in superior caloric and protein delivery compared with conventional physician-directed nutritional management without a standardized protocol for achieving prescribed targets in critically ill surgical ICU patients, while maintaining safety with respect to RFS and gastrointestinal complications.

## 2. Materials and Methods

### 2.1. Study Design and Setting

**Figure 1:**
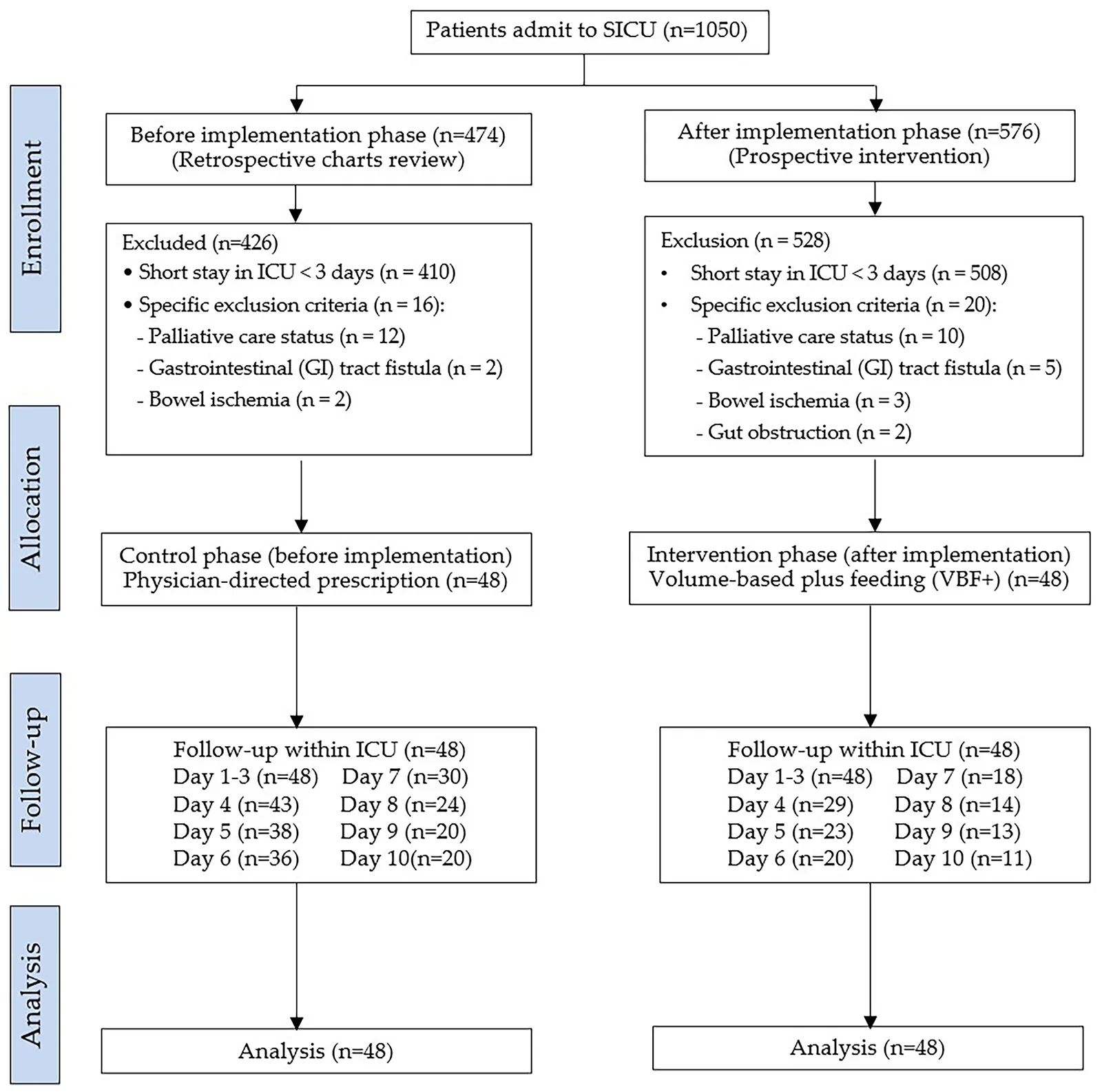
Flow diagram of study participants.

The study was conducted using ambispective before-and-after design, comprising a retrospective (before intervention) phase and a prospective (post-intervention) phase, in the Surgical Intensive Care Unit (SICU) of Maharaj Nakorn Chiang Mai Hospital, a university-affiliated tertiary care center in northern Thailand (Faculty of Medicine, Chiang Mai University). The retrospective control phase included patients treated before implementation, from 1 January 2023 to 19 March 2024. The prospective implementation phase was initiated after approval from the Institutional Review Board and was conducted in two periods: from 20 March 2024 to 19 March 2025, and from 6 May 2025 to 19 March 2026. Consistent with the a priori sample size calculation (Section 2.2), enrollment in each phase continued until 48 evaluable patients were reached; patients who met eligibility criteria after this target was achieved within a given phase’s enrollment window were not included in the analysis (Figure 1). Patient enrollment was suspended between 19 March 2025 and 6 May 2025 because renewal of the Institutional Review Board (Ethics Committee) approval for continuation of the study was delayed beyond the original approval period; enrollment resumed once the renewed approval was granted. This interruption was administrative in nature and was unrelated to patient safety, protocol conduct, or the clinical characteristics of enrolled patients. The study was approved by the institutional ethics committee (study code: SUR-2566-0439, Research ID: 439), and written informed consent was obtained from patients, legal guardians, or designated power of attorney prior to enrolment during intervention phase.

The study comprised two sequential phases: a control phase (before implementation) and an intervention phase (after implementation). Patients in both phases underwent the same refeeding-risk assessment and were assigned to the same categories of feeding strategy. The intervention phase additionally implemented the standardized Volume-Based Feeding Plus (VBF+) protocol, which incorporated refeeding-risk-guided caloric advancement and, a standardized semi-elemental enteral formula, volume-based compensation for feeding interruptions.

### 2.2. Sample Size Calculation

The sample size was calculated a priori for a two-sample means test using Satterthwaite’s *t* test assuming unequal variances, based on the primary outcome of daily caloric delivery (% of target). Estimates of the mean and standard deviation for each group were derived from a previously published volume-based feeding trial, in which the volume-based feeding group received 92.9% ± 16.8% of goal caloric requirements compared with 80.9% ± 18.9% in the rate-based feeding group (*p* = 0.01) [5]. Using these estimates (m1 = 92.9, sd1 = 16.8; m2 = 80.9, sd2 = 18.9; delta = −12.0 percentage points), a two-sided alpha of 0.05, and a target power of 0.90, the required sample size was calculated as 48 patients per group (*N* total = 96).

### 2.3. Participants

Adult patients (aged ≥20 years) admitted to the SICU and expected to require enteral nutrition (EN) for at least 72 h were eligible. Patients were excluded if they were pregnant, receiving palliative care, or had conditions precluding enteral feeding, including short bowel syndrome, untreatable intestinal obstruction, gastrointestinal fistula, bowel ischemia, severe intra-abdominal hypertension (grade > 3), radiation enteritis, ulcerative colitis, or chronic intestinal failure.

Enteral feeding was discontinued if a gastric residual volume (GRV) > 300 mL was observed on two consecutive 4-h assessments, or if vomiting, regurgitation, or aspiration occurred.

### 2.4. Risk Stratification and Feeding Strategy Assignment

**Figure 2:**
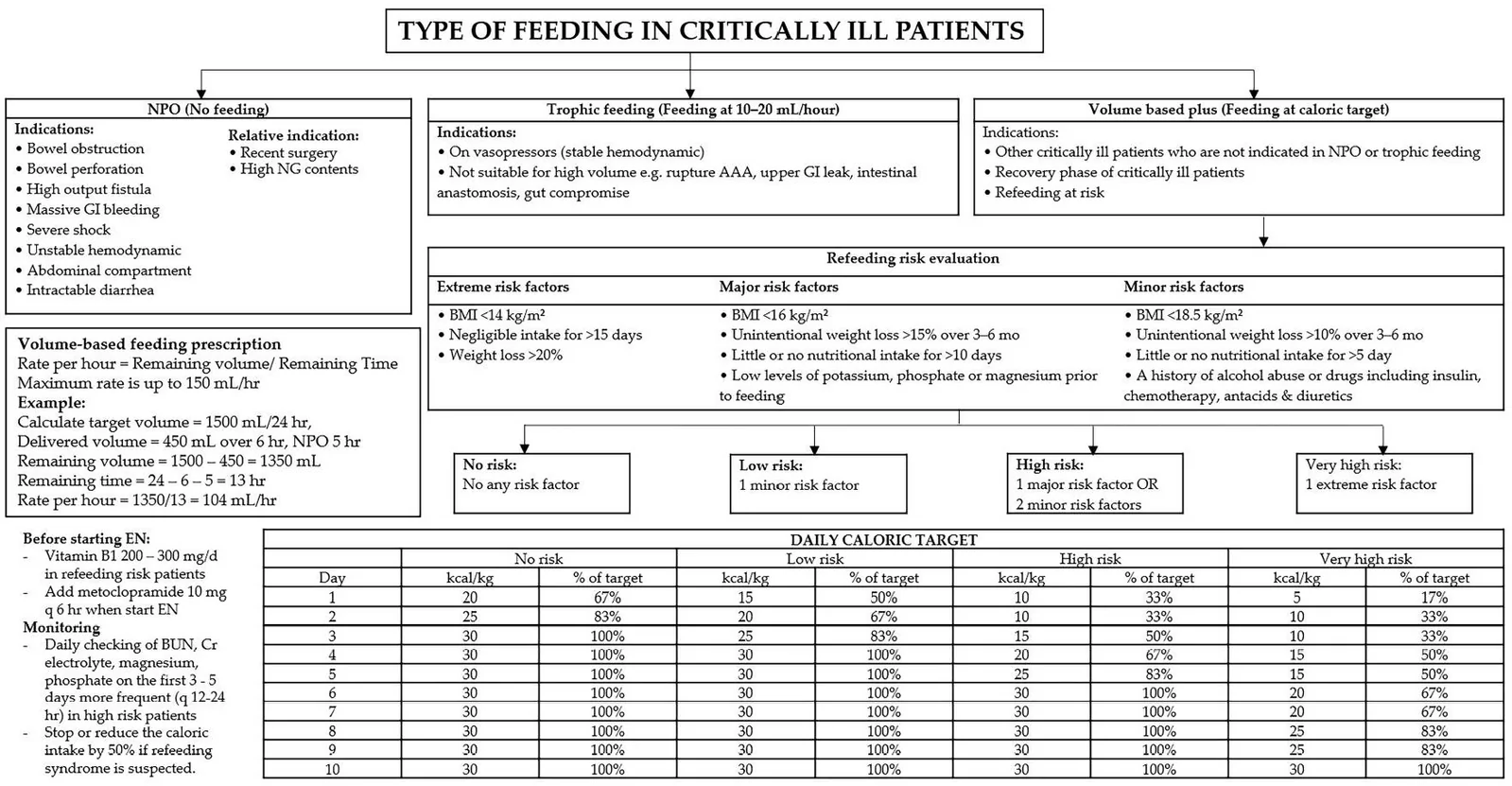
Clinical flowchart for nutritional pathway selection, refeeding syndrome risk stratification, and the 10-day daily caloric progression matrix in critically ill patients.

Before initiation of enteral nutrition, all patients underwent a standardized assessment for refeeding syndrome risk, with the classification logic conceptually informed by the criteria of Friedli et al. (2018) [10], while the specific thresholds, caloric progression matrix, and figure design were developed independently by the study authors for this protocol. Patients were classified into four refeeding-risk categories: no risk, low risk, high risk, and very high risk, according to the presence of minor, major, or extreme risk factors as defined in the protocol (Figure 2). Although a very-high-risk category was defined in the protocol, no patients met these criteria during the study period; all enrolled patients therefore fell into the no-risk, low-risk, or high-risk categories.

In addition to refeeding-risk stratification, feeding strategy was also determined by hemodynamic status. Patients receiving vasopressor support were eligible for trophic feeding if hemodynamically stable, defined as a norepinephrine-equivalent vasopressor dose of 0.14–0.3 μg/kg/min [11]; patients requiring vasopressor doses above this threshold were maintained nil per os (NPO) until the dose decreased below the threshold or vasopressor support was discontinued.

Daily caloric targets were progressively advanced over a 10-day period according to the assigned refeeding-risk category, up to a maximum of 30 kcal/kg/day (100% of target), as detailed in Figure 2. For the no-risk group, caloric delivery targets were set at 65% (20 kcal/kg/day) on Day 1, 80% (25 kcal/kg/day) on Day 2, and 100% (30 kcal/kg/day) from Day 3 onwards. For patients with low refeeding risk, targets advanced from 50% on Day 1 to 100% by Day 4. For those with high refeeding risk, a more gradual advancement was employed, reaching 100% by Day 10. No patient underwent indirect calorimetry during the study period.

Patients classified as low or high refeeding risk received prophylactic thiamine (vitamin B1) supplementation at 200–300 mg/day throughout the advancement period. In addition, intravenous metoclopramide (10 mg every 6 h) was routinely initiated at the start of enteral nutrition to facilitate gastric emptying and optimize gastrointestinal tolerance.

The classification logic of the refeeding-risk-evaluation panel was conceptually adapted from the risk-stratification framework of Friedli et al. (2018) [10]; the specific risk thresholds used in our cohort, the visual design and layout of the figure, the nutritional pathway selection flowchart, and the day-by-day caloric progression matrix were developed originally by the study authors for the VBF+ protocol and do not reproduce any figure, table, or graphical element from the original publication. Hemodynamic stability for trophic feeding eligibility was defined as a norepinephrine-equivalent vasopressor dose ≤0.3 μg/kg/min (with a suggested safe range of 0.14–0.3 μg/kg/min based on existing evidence) [11]; patients above this threshold were maintained NPO.

### 2.5. Intervention and Control Protocols

Control Phase (Before): Patients received enteral nutrition according to usual care. The institution had no written or customary nutritional target (e.g., no standard institutional kcal/kg/day reference range or departmental convention) during this period. Caloric targets and feeding advancement were determined entirely by the treating physician without a standardized protocol or a predefined strategy to compensate for feeding interruptions. The same refeeding-risk assessment criteria were applied to all control-phase patients, and the same risk-stratified caloric advancement framework was used as a reference; however, targets were implemented at the physician’s discretion without a mandated volume-based compensation strategy. As in the intervention phase, patients classified as low or high refeeding risk received the same prophylactic thiamine supplementation and routine intravenous metoclopramide (10 mg every 6 h) regimen; only the volume-based compensation algorithm and the enteral formula differed between phases.

Intervention Phase (VBF+ Protocol): Patients were managed using the VBF+ protocol, a standardized, refeeding-risk-guided feeding strategy incorporating progressive caloric advancement and volume-based compensation for feeding interruptions, and a standardized semi-elemental enteral formula. Initial daily caloric delivery targets were determined according to body weight and the assigned refeeding-risk category, following the predefined progression matrix described in Section 2.3 and illustrated in Figure 2.

A key component of the protocol was the use of a compensatory rate algorithm, which enabled bedside nurses to adjust enteral nutrition infusion rates following interruptions in order to recover the prescribed volume within the remaining feeding time of a 24-h cycle. A maximum infusion rate of 150 mL/hour was applied as a safety limit; if the required compensatory rate exceeded this threshold, physicians were prompted to adjust the caloric density of the formula instead (Table S1). In the pre-implementation phase, enteral nutrition was provided using a standard generic polymeric formula, whereas in the post-implementation phase a semi-elemental formula (Peptamen, Nestle Health Science) was administered. The switch to a semi-elemental formula was implemented as an integral component of the VBF+ bundle alongside the compensatory algorithm and risk-stratified caloric advancement, rather than as an independent variable; VBF+ should therefore be understood as a multicomponent nutritional care bundle.

Patients classified as low or high refeeding risk received prophylactic thiamine (200–300 mg/day) and intravenous metoclopramide (10 mg every 6 h) from the initiation of enteral nutrition, identical to the control phase. Gastric residual volume was assessed every 4 h in accordance with the standardized nurse-led enteral feeding bundle.

### 2.6. Outcomes

Primary outcomes: (1) Median daily caloric delivery expressed as percentage of the prescribed target; (2) mean daily protein delivery expressed as grams per kilogram body weight per day (g/kg/day). Daily protein intake was recorded in grams per day and normalized to body weight for analysis.

Secondary outcomes: Incidence of gastrointestinal complications (watery diarrhea > 5 times/day, gastric intolerance defined as GRV > 300 mL, vomiting, regurgitation, and aspiration); incidence of RFS-related electrolyte imbalances; and clinical outcomes (ICU length of stay and in-hospital mortality).

### 2.7. Statistical Analysis

Continuous variables were described as mean ± standard deviation (SD) or median with interquartile range (IQR), as appropriate. Categorical variables were summarized as frequency and percentage. Between-group comparisons used Student’s *t* test or the Mann–Whitney U test for continuous variables, and the chi-squared or Fisher’s exact test for categorical variables.

Daily caloric delivery and protein delivery were summarized descriptively according to refeeding-risk category (no risk, low risk, and high risk) within the intervention and control groups for each ICU day and across the entire observation period. Percent of prescribed target was used as the primary caloric delivery metric because targets are risk-stratified and advance progressively by design, so percent-of-target directly reflects whether each patient’s individually prescribed, safety-adjusted target was met, which absolute intake alone cannot indicate; absolute caloric delivery (kcal/kg/day) was additionally summarized descriptively to allow percent-of-target values to be interpreted alongside absolute intake. Caloric delivery was presented as median (IQR), whereas protein delivery was summarized as mean ± SD according to their respective distributions. For these descriptive subgroup tables, statistical comparisons were performed between the intervention and control groups for each study day irrespective of refeeding-risk category, using the Mann–Whitney U test for caloric delivery and Welch’s two-sample *t* test for protein delivery. Refeeding-risk categories were included solely to describe the distribution of nutritional delivery within each treatment group and were not compared statistically.

Prior to model fitting, the distribution of outcome variables was assessed at two levels. At the observation level (daily values, n = 627), caloric delivery showed substantial right skewness (skewness = 2.11, Shapiro–Wilk *p* < 0.001), whereas protein delivery was approximately symmetric (skewness = −0.13) (Figure S1). At the patient level, individual summaries were derived accordingly: median of daily values for caloric delivery and mean of daily values for protein delivery. The choice of median versus mean as the patient-level summary statistic was based on the skewness of the observation-level distribution rather than on the Shapiro–Wilk test, given the well-known tendency of the latter to reject normality in large samples (n = 627) even when distributional departures are clinically negligible. The patient-level distribution of median caloric delivery remained non-normal (Shapiro–Wilk *p* < 0.001, skewness = 0.98), whereas mean protein delivery was approximately normal (Shapiro–Wilk *p* = 0.414, skewness = 0.06) (Figure S2).

To evaluate the independent association between the feeding protocol and nutritional outcomes, multivariable regression models were constructed. For caloric delivery, given the non-normal patient-level distribution, a median regression model (quantile regression at τ = 0.5) with bootstrap standard errors (1000 replications) was applied to the patient-level median. For protein delivery, a multivariable linear regression model was applied to the patient-level mean. The primary exposure variable was study group (VBF+ vs. control). Both models were adjusted a priori for nutritional assessment score (NAF), APACHE II score, and Charlson Comorbidity Index (CCI), selected on the basis of clinical relevance and potential associations with nutritional delivery. Multicollinearity was assessed using generalized variance inflation factors (GVIF), with adjusted GVIF^(1/(2 × df))^ values < 2.0 considered acceptable.

To account for repeated daily measurements and intra-patient correlation, linear mixed-effects models were fitted with fixed effects of study group, ICU day, and their interaction (group × day), and a random intercept for each patient. Both outcomes were modeled on their original scales without transformation. For caloric delivery, the linear mixed-effects model was used to characterize longitudinal trajectories and interaction patterns rather than to estimate the primary intervention effect, because the outcome remained substantially non-normally distributed at the observation level (skewness = 2.11). The primary estimate of treatment effect for caloric delivery was therefore obtained from the multivariable median regression model. Residuals from the caloric delivery mixed-effects model exhibited moderate skewness (skewness = 1.39), whereas those from the protein delivery model were approximately symmetric (skewness = −0.04), supporting the adequacy of the mixed-effects analysis for evaluating longitudinal trends in protein delivery. These models assume that data are missing at random conditional on the random intercept; because a substantial proportion of patients exited the analysable sample before Day 10 through ICU discharge or death, this assumption may not fully hold, and the results should be interpreted with this caveat (see Section 3.3).

To address potential confounding inherent to the before-and-after study design, propensity scores were estimated using a logistic regression model including age, sex, APACHE II score, Charlson Comorbidity Index (CCI), and refeeding-risk category. Two propensity score-based approaches were applied: (1) inverse probability of treatment weighting (IPTW) using stabilized weights truncated at the 1st and 99th percentiles, followed by survey-weighted linear regression; (2) 1:1 nearest-neighbor propensity score matching (PSM) without replacement, followed by median regression (quantile regression at τ = 0.5) for caloric delivery and linear regression for protein delivery in the matched cohort. Covariate balance was evaluated using standardized mean differences (SMDs), with an absolute SMD < 0.10 indicating acceptable balance. Love plots were generated to illustrate covariate balance before and after adjustment (Figure S4).

All analyses were performed in R version 4.3 (R Foundation for Statistical Computing). A two-sided *p* value < 0.05 was considered statistically significant.

## 3. Results

### 3.1. Baseline Characteristics

A total of 96 critically ill surgical patients were included, with 48 patients in each study phase. Baseline characteristics are summarized in Table 1.

Overall, the two groups were broadly comparable with respect to demographic characteristics, nutritional status, and illness severity, including age, sex, BMI, nutritional assessment, and APACHE II and SOFA scores (all *p* > 0.05). Refeeding risk stratification was also similar between groups, and no patients were classified as very high risk in either phase.

However, two clinically relevant imbalances were observed. The Charlson Comorbidity Index was higher in the intervention group, indicating a greater burden of chronic comorbidities, whereas the SAPS II score was higher in the control group, suggesting greater acute illness severity at ICU admission.

In addition, the distribution of feeding strategies differed significantly between phases, with a higher proportion of trophic feeding and a lower proportion of patients without feeding restrictions (“no any risk”) in the intervention group (both *p* < 0.05). This distribution difference likely reflects the evolution of clinical practice over the study period; regardless of its origin, the imbalance in feeding strategy between phases was addressed through multivariable adjustment and propensity score analyses.

### 3.2. Primary Outcomes: Nutritional Delivery

After multivariable adjustment, the VBF+ protocol was independently associated with substantially higher caloric and protein delivery compared with the control group (Table 2). For caloric delivery, the effect corresponded to an increase of 31.2 percentage points (95% CI 17.6 to 44.8, *p* < 0.001). For protein delivery, the VBF+ protocol was associated with an increase of 0.168 g/kg/day (95% CI 0.040 to 0.297, *p* = 0.011). Nutritional status assessed by the nutritional assessment form (NAF) was also independently associated with caloric delivery: both moderate malnutrition (β = 24.2 percentage points, 95% CI 1.55 to 46.8, *p* = 0.037) and severe malnutrition (β = 25.4 percentage points, 95% CI 1.86 to 49.0, *p* = 0.035) were associated with higher caloric delivery compared with normal–mild malnutrition, potentially reflecting more aggressive nutritional management in patients with greater nutritional deficits. APACHE II score and Charlson Comorbidity Index were not significantly associated with either outcome. No evidence of multicollinearity was identified in either model (all adjusted GVIF^(1/(2 × df))^ < 2.0; Table S4).

Daily nutritional delivery stratified by refeeding-risk category is summarized in Table S2 (caloric delivery, both percent of target (Table S2A) and absolute intake in kcal/kg/day (Table S2B)) and Table S3 (protein delivery). Consistent with the protocol-defined caloric advancement schedule, caloric delivery varied across refeeding-risk categories during the early ICU period, whereas protein delivery showed only modest differences among categories.

### 3.3. Longitudinal Trajectories of Nutritional Delivery

**Figure 3:**
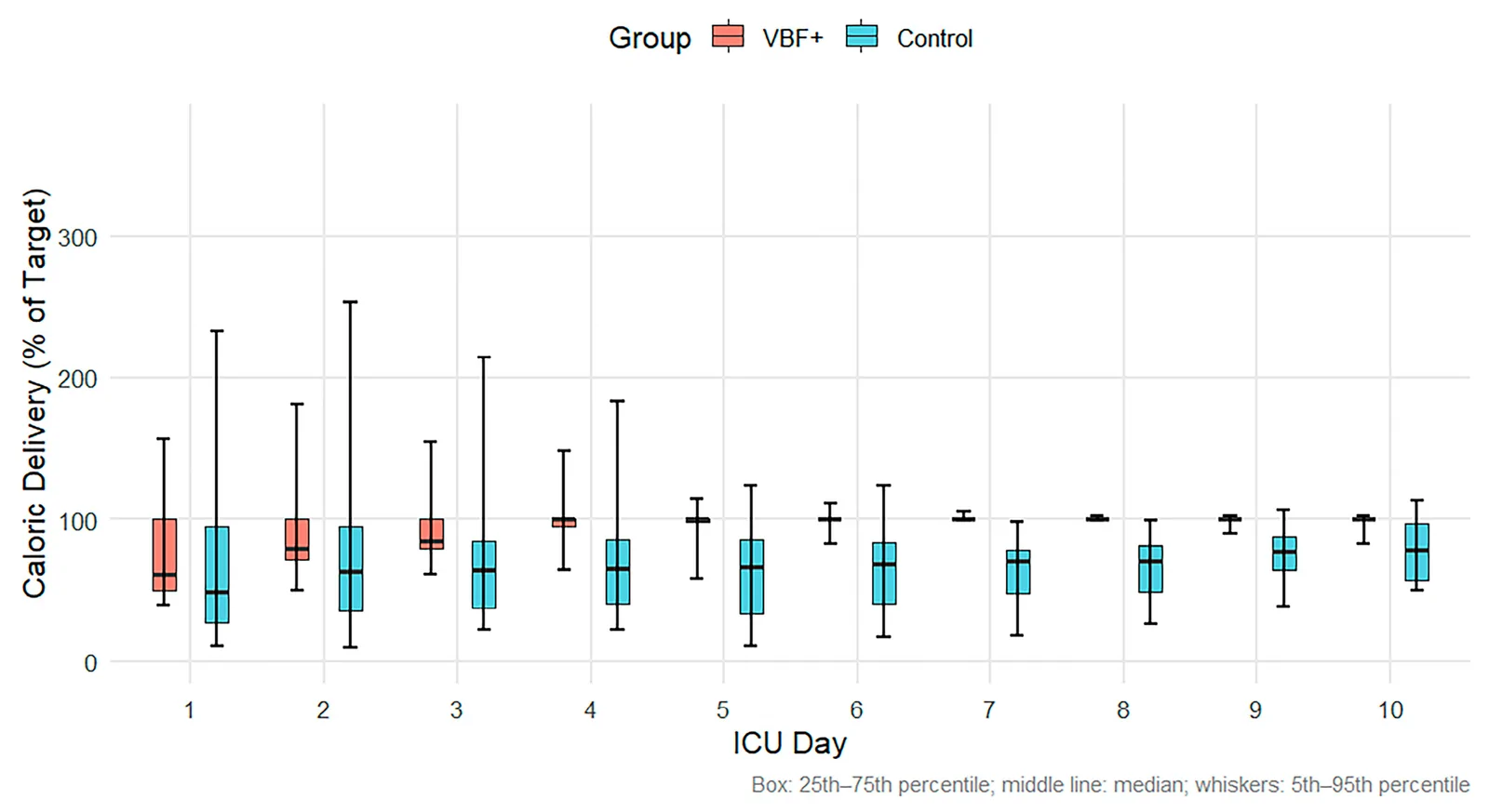
Daily caloric delivery (% of target) in the intervention and control groups over the first 10 ICU days.

**Figure 4:**
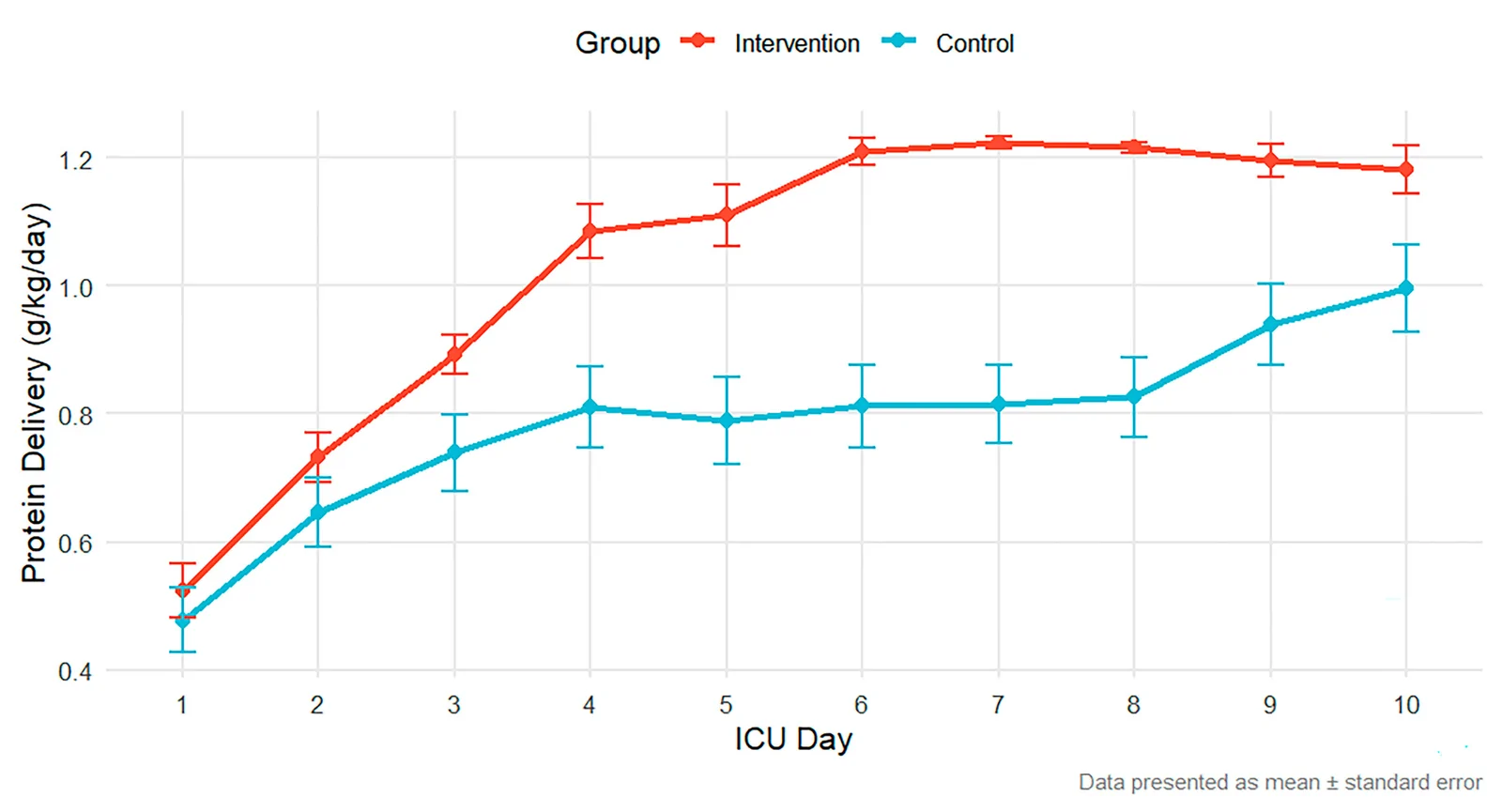
Daily protein delivery (g/kg/day) in the intervention and control groups over the first 10 ICU days.

Longitudinal mixed-effects models were used to evaluate temporal changes in nutritional delivery during the first 10 ICU days (Figure 3 and Figure 4). For caloric delivery, neither the overall time effect (β = 0.85 percentage points per day, 95% CI −0.95 to 2.64, *p* = 0.356) nor the group-by-time interaction reached statistical significance, although the point estimate suggested a steeper increase in caloric delivery in the intervention group (interaction β = +2.10 percentage points per day relative to the control group, 95% CI −0.20 to 4.40, *p* = 0.073).

In contrast, protein delivery increased significantly over time in both groups (β = 0.080 g/kg/day per day, 95% CI 0.066–0.093, *p* < 0.001). A significant group-by-time interaction was also observed, indicating that the rate of increase was 0.035 g/kg/day per day greater in the intervention group than in the control group (interaction β = +0.035 g/kg/day per day, 95% CI 0.018–0.053, *p* < 0.001). Although the overall between-group difference was not statistically significant (β = 0.050 g/kg/day, 95% CI −0.063 to 0.162, *p* = 0.383), the significant interaction indicates that the VBF+ protocol primarily accelerated the attainment of protein delivery targets over time rather than producing a constant difference throughout the study period.

The analysable sample size declined substantially over the 10-day observation period, from 48 to 11 patients in the intervention group and from 48 to 20 patients in the control group, predominantly because of ICU discharge and death. This attrition was more pronounced and occurred earlier in the intervention group, consistent with the numerically shorter ICU length of stay and lower mortality observed in this group (Figure 1). Because exit from observation was driven by clinical improvement (discharge) or death rather than occurring at random, the later time points in the mixed-effects models are based on a progressively smaller and differentially selected subset of patients in each group, which should be considered when interpreting the group-by-time interaction for protein delivery.

Data are presented as boxplots (median, interquartile range; whiskers indicate the 5th–95th percentiles). Longitudinal trends were analyzed using a linear mixed-effects model with a random intercept for patients. Neither the overall time effect nor the group-by-time interaction reached statistical significance for caloric delivery (*p* = 0.073 for the interaction).

Data are presented as mean ± standard error. Longitudinal trends were analyzed using a linear mixed-effects model with a random intercept for patients. Protein delivery increased significantly over time in both groups, with a significant group-by-time interaction indicating a more rapid increase in the intervention group than in the control group.

### 3.4. Safety and Complications

Complication rates are summarized in Table 3. Overall, the VBF+ protocol was not associated with an increased risk of gastrointestinal or metabolic complications and was, in some aspects, associated with improved tolerance.

The VBF+ group demonstrated significantly lower rates of gastric intolerance and diarrhea. Gastric intolerance (GRV > 300 mL) occurred in 2.08% of patients in the intervention group compared with 16.67% in the control group (*p* = 0.031). Watery diarrhea (>5 times/day) was observed only in the control group (12.50% vs. 0%; *p* = 0.026). Other gastrointestinal events, including vomiting and regurgitation, were numerically less frequent in the intervention group but did not reach statistical significance. Aspiration rates were similar between groups (6.25% vs. 12.50%; *p* = 0.486).

There were no significant between-group differences in refeeding-related biochemical abnormalities. A decrease in serum phosphate of >30% or to <0.6 mmol/L occurred in 50.0% of at-risk patients in the intervention group and 40.0% in the control group (*p* = 0.691). Critical electrolyte abnormalities were rare and occurred at identical rates in both groups (2.08% vs. 2.08%; *p* = 1.000). These findings indicate that the VBF+ protocol did not increase the metabolic risk of refeeding syndrome.

### 3.5. Clinical Outcomes

Exploratory clinical outcomes are presented in Table 4. Although the study was not powered to detect differences in clinical endpoints, trends favored the VBF+ group. Clinical improvement at ICU discharge or study completion was more frequent in the intervention group (77.1% vs. 64.6%), while in-hospital mortality was numerically lower (6.25% vs. 14.58%); however, these differences did not reach statistical significance (overall status comparison *p* = 0.547).

ICU length of stay was similar between groups (median 12.0 days [IQR 9.0–19.5] vs. 13.0 days [IQR 7.0–21.0]; *p* = 0.940).

These findings should be interpreted with caution given the limited sample size, and they are consistent with prior studies in the literature indicating that improvements in nutritional delivery do not necessarily translate into detectable differences in mortality or length of stay in small cohorts.

**Table 4:** Clinical exploratory outcomes.

Outcome	Total (n = 96)	Intervention (n = 48)	Control (n = 48)	*p*-Value
Clinical status at discharge/study end, n (%)				0.547
Improved	68	37 (77.1)	31 (64.6)	
Stable	6	3 (6.3)	3 (6.3)	
Worsening	1	0 (0)	1 (2.1)	
Died after ICU discharge	10	3 (6.3)	7 (14.6)	
Expected death after discharge	11	5 (10.4)	6 (12.5)	
ICU length of stay, median (IQR) days		12.0 (9.0–19.5)	13.0 (7.0–21.0)	0.940

IQR, interquartile range. Clinical status categories were defined as follows: improved (clinical recovery or discharge readiness), stable (no significant change), worsening (clinical deterioration), died (in-hospital mortality), and expected death (palliative or end-of-life care).

### 3.6. Propensity Score-Based Sensitivity Analyses

The distribution of propensity scores demonstrated adequate overlap between the intervention and control groups (Figure S3), supporting the positivity assumption. Before adjustment, several baseline characteristics showed meaningful imbalance, particularly age and Charlson Comorbidity Index, consistent with the non-randomized before-and-after study design.

Both propensity score approaches substantially improved covariate balance (Figure S4). After IPTW, all baseline covariates achieved excellent balance, with absolute standardized mean differences below 0.10. Similarly, 1:1 nearest-neighbor propensity score matching with a caliper width of 0.2 standard deviations of the logit of the propensity score yielded a matched cohort of 72 participants (36 matched pairs), in which all baseline covariates also achieved acceptable balance.

The propensity score-based analyses produced findings consistent with the primary analysis (Table S5). After IPTW adjustment, the VBF+ group remained associated with significantly higher caloric delivery (β = 18.95 percentage points, 95% CI 5.24–32.66; *p* = 0.007) and significantly higher protein delivery (β = 0.139 g/kg/day, 95% CI 0.028–0.250; *p* = 0.015). In the propensity score-matched cohort, caloric delivery (β = 29.30 percentage points, 95% CI 18.50–41.00; *p* = 0.001) and protein delivery (β = 0.162 g/kg/day, 95% CI 0.040–0.283; *p* = 0.010) also remained significantly higher in the VBF+ group.

Across all analytical approaches, the estimated effect of the VBF+ protocol on caloric delivery remained consistently favorable, with adjusted effect estimates ranging from 18.95 to 31.2 percentage points. The consistency in both direction and magnitude across multivariable regression, mixed-effects modeling, IPTW, and PSM analyses provides additional support for the robustness of the observed improvement in caloric delivery.

Nevertheless, because this was a single-center before-and-after observational study, the propensity score analyses should be interpreted as sensitivity analyses supporting the robustness of the primary findings rather than as evidence of a definitive causal effect.

## 4. Discussion

The principal findings of this study are twofold. First, descriptive analyses showed differences in caloric delivery across refeeding-risk categories, whereas protein delivery was generally similar among risk groups. However, because refeeding-risk categories were presented for descriptive purposes only and were not formally compared, these findings should be interpreted as illustrating the distribution of nutritional delivery rather than demonstrating independent effects of refeeding risk. Second, implementation of the VBF+ protocol was independently associated with substantially improved nutritional delivery, resulting in a 31.2 percentage point increase in caloric delivery and a 0.168 g/kg/day increase in protein intake after adjustment for nutritional assessment, APACHE II score, and CCI. These findings were further supported by propensity score-based sensitivity analyses, whereas the longitudinal mixed-effects models primarily characterized temporal patterns of nutritional delivery rather than serving as the primary estimates of intervention effect. Notably, the intervention group had a significantly higher Charlson Comorbidity Index than the control group, which would be expected to bias nutritional outcomes toward the null; the finding that VBF+ was nonetheless associated with higher nutritional delivery, with CCI itself not independently associated with either outcome after adjustment, argues against comorbidity burden as an alternative explanation for this benefit.

From a clinical perspective, the magnitude of improvement in caloric delivery is substantial. Unlike reporting target achievement rates alone, the use of absolute between-group differences (percentage points) provides a more interpretable measure of intervention effect, particularly in non-randomized designs. Previous studies of volume-based feeding have primarily focused on achieving higher proportions of prescribed targets, typically reporting improvements in the range of 10–25 percentage points [5,6,12]. The effect size observed in the present study is therefore at the upper end of reported improvements, suggesting that the integration of structured risk stratification and protocolized decision-making may enhance the effectiveness of volume-based strategies [13,14].

Although patients classified as low risk generally exhibited higher caloric delivery than other risk categories during the early ICU period, these subgroup summaries primarily reflect the predefined caloric advancement strategy incorporated into the protocol. Because formal statistical comparisons among refeeding-risk categories were not performed, these findings should be viewed as descriptive evidence that the protocol successfully implemented differential caloric progression according to refeeding risk. Protein delivery remained relatively consistent across risk categories, suggesting that adequate protein provision was maintained despite different caloric advancement schedules.

An important methodological aspect of this study is the approach to protein outcome assessment. Rather than expressing protein delivery as a percentage of target—which may vary across clinicians and settings—we quantified intake as g/kg/day, aligning directly with guideline recommendations (typically 1.2–2.0 g/kg/day in critically ill patients) [15]. Both multivariable regression and PSM analyses demonstrated a significant improvement in protein delivery with VBF+, whereas the mixed-effects model demonstrated a significant group-by-time interaction, indicating that protein delivery increased more rapidly over time in the intervention group despite no significant overall between-group difference at baseline. This suggests that the benefit of the protocol lies not only in higher overall intake but also in earlier attainment of adequate protein provision, which may be clinically relevant given the association between early protein delivery and preservation of lean body mass and improved recovery [16,17]. However, this interpretation should be tempered by the marked and differential decline in sample size over the 10-day window (48 to 11 in the intervention group vs. 48 to 20 in the control group), driven largely by discharge and death rather than random loss to follow-up. Because such attrition is not missing-at-random, later time points disproportionately represent patients who remained critically ill for longer, and this changing cohort composition—rather than a true treatment effect—could partly explain the steeper protein trajectory observed in the intervention group. The group-by-time interaction for protein delivery should therefore be interpreted as hypothesis-generating.

Concerns have been raised that volume-based feeding, particularly with compensatory rate adjustments, may increase gastrointestinal intolerance. Our findings do not support this concern. The VBF+ group demonstrated significantly lower rates of gastric intolerance and diarrhea, despite higher nutritional delivery. This likely reflects two components of the VBF+ bundle. First, minimizing feeding interruptions and compensating for lost volume—the core function of the volume-based algorithm itself—may reduce variability in nutrient delivery, which has been hypothesized to adversely affect gastrointestinal function [18]. Second, the semi-elemental formula administered as part of VBF+ is more readily absorbed and empties from the stomach faster than the standard polymeric formula used in the control phase, and may independently have contributed to improved tolerance. Because the prokinetic regimen, trophic feeding criteria, and refeeding-risk assessment were identical in both phases, they cannot account for the between-group difference. The present design cannot separate the relative contribution of the algorithm from that of the formula to this benefit.

Importantly, improved nutritional delivery was not associated with increased metabolic risk. The incidence of refeeding-related electrolyte abnormalities was low and comparable between groups, indicating that the risk-stratified caloric ramp-up strategy effectively mitigated the risk of refeeding syndrome. By tailoring initial caloric provision according to refeeding risk and incorporating prophylactic thiamine supplementation, the protocol appears to safely reconcile early nutritional adequacy with metabolic stability [10].

The use of complementary analytical approaches strengthens the robustness of these findings. The primary multivariable regression models provided the principal estimates of treatment effect after adjustment for baseline nutritional status, disease severity, and comorbidity burden. The linear mixed-effects models complemented these analyses by characterizing longitudinal trajectories, demonstrating no significant group-by-time interaction for caloric delivery but a significant interaction for protein delivery, indicating a faster increase in protein intake in the intervention group over time. Propensity score-based sensitivity analyses achieved excellent covariate balance after both IPTW and propensity score matching and produced effect estimates consistent with the primary analyses. Together, these complementary methods provide convergent evidence supporting the robustness of the observed nutritional benefits while acknowledging the inherent limitations of causal inference in a before-and-after observational design [19].

Safety and complication outcomes in our study suggest a potential benefit of the intervention protocol. Patients in the intervention group had significantly lower rates of watery diarrhea and gastric intolerance compared with those in the control group. However, these findings should be interpreted with caution, because VBF+ combined the volume-based compensatory algorithm with a switch to a semi-elemental enteral formula as part of a single implementation bundle. The intervention group received a semi-elemental diet, which is known to be more readily absorbed and to have faster gastric emptying than standard polymeric formulas, whereas the control group received a conventional generic formula. This difference in formula composition is therefore a component of the VBF+ bundle rather than an independent variable, and the present before-and-after design cannot determine the relative contribution of the algorithm versus the formula to the observed reduction in gastrointestinal adverse events. Accordingly, these gastrointestinal tolerance findings should be regarded as hypothesis-generating pending confirmation in a design that varies the algorithm and formula independently. The same caveat applies in principle to the primary outcomes of caloric and protein delivery: because formula type could not be statistically separated from group assignment, we cannot exclude a contribution of the formula itself (e.g., its caloric density or tolerability) to the observed improvements in nutritional delivery.

Several limitations should be acknowledged. First, the before-and-after design is susceptible to temporal confounding, including changes in clinical practice or case mix during the enrollment, and to asymmetric data ascertainment between the retrospective control phase and the prospective intervention phase, which could affect data completeness or accuracy independently of any true effect of the VBF+ protocol. Second, blinding was not feasible, which may have influenced clinician or nursing behavior. Third, the single-center setting may limit generalizability. Fourth, the sample size was insufficient to detect differences in clinical endpoints such as mortality or length of stay. Fifth, because the VBF+ protocol bundled the volume-based compensatory algorithm with a change in enteral formula, the independent contribution of each component to the observed caloric delivery, protein delivery, and gastrointestinal outcomes cannot be determined from this design. Because formula type was completely aligned with study group assignment, it is perfectly collinear with the intervention variable and therefore could not be entered as a covariate in the multivariable regression, mixed-effects, or propensity-score models; conventional statistical adjustment cannot separate the effect of the algorithm from that of the formula. Consequently, all between-group differences reported in this study—not only the gastrointestinal findings—should be interpreted as attributable to the VBF+ bundle as implemented, rather than to the compensatory algorithm in isolation. Sixth, the analysable sample size for the longitudinal mixed-effects models declined substantially by Day 10 (from 48 to 11 patients in the intervention group and from 48 to 20 in the control group), predominantly through ICU discharge and death. Because this attrition was differential between groups and not missing-at-random, it may have influenced the group-by-time interaction estimates, particularly for protein delivery, and the linear mixed-effects models’ assumption of data missing at random conditional on the random intercept may not be fully satisfied. Finally, because caloric delivery remained non-normally distributed even after patient-level aggregation, longitudinal mixed-effects analyses for this outcome should be interpreted as descriptive assessments of temporal trends rather than definitive estimates of intervention effect.

Future research should focus on confirming these findings in multicenter randomized controlled trials to establish causal inference and generalizability. In addition, studies incorporating body composition assessment (e.g., muscle mass or function) and longer-term outcomes are needed to determine whether improvements in early nutritional delivery translate into meaningful patient-centered benefits. The integration of automated feeding algorithms and real-time decision support may further enhance protocol adherence and scalability across diverse ICU settings.

## 5. Conclusions

In this before-and-after study, implementation of the VBF+ protocol was associated with significantly improved caloric and protein delivery, along with lower rates of gastrointestinal intolerance. Because VBF+ was implemented as a bundle combining the volume-based algorithm with a semi-elemental formula, the gastrointestinal findings should be regarded as hypothesis-generating. These results support further evaluation of a risk-stratified, volume-based enteral nutrition protocol in multicenter randomized trials.

## Figures and Tables

**Table 1:** Characteristics of included studies.

Characteristics	Intervention Group (n = 48)	Control Group (n = 48)	*p*-Value
Age, mean ± SD	64.97 ± 18.48	62.63 ± 18.01	0.216
Sex, n (%)			0.675
Male	31 (64.58)	28 (58.33)	
Female	17 (35.42)	20 (41.67)	
Type of refeeding risk, n (%)			0.329
No risk	29 (60.42)	30 (62.50)	
Low risk	7 (14.58)	11 (22.92)	
High risk	12 (25.00)	7 (14.58)	
Very high risk	0 (0)	0 (0)	
BMI, mean ± SD	22.16 ± 4.70	22.05 ± 4.32	0.900
Weight, mean ± SD	57.34 ± 14.58	59.29 ± 13.60	0.376
Nutritional assessment “NAF”, n (%)			0.370
Normal–Mild malnutrition	8 (16.67)	9 (18.75)	
Moderate malnutrition	26 (54.17)	31 (64.58)	
Severe malnutrition	14 (29.17)	8 (16.67)	
Priority, n (%)			0.638
Priority 1 (reversible instability)	40 (83.33)	42 (87.50)	
Priority 2 (monitoring)	1 (2.08)	2 (4.17)	
Priority 3 (irreversible instability)	7 (14.58)	4 (8.33)	
Charlson Comorbidity Index (CCI), median (IQR)	5 (2.5–7)	3.5 (1.5–5)	0.010
APACHE II score, mean ± SD	23.13 ± 6.57	23.15 ± 6.11	0.987
SOFA score, mean ± SD	7.00 ± 3.26	7.46 ± 4.32	0.559
SAPS II score, mean ± SD	47.85 ± 12.43	41.79 ± 14.72	0.032

“No any risk” refers to patients without refeeding syndrome risk or hemodynamic/anatomical contraindications, corresponding to eligibility for full-volume feeding (volume-based feeding strategy). Priority Levels for ICU Admission: 1 = patients with unstable vital signs and reversible conditions (the cause is treatable); 2 = patients at risk of vital sign instability who require intensive monitoring in the ICU; 3 = patients with unstable vital signs where the underlying disease is not fully curable (chronic or irreversible conditions). IQR, interquartile range; BMI, body mass index; NAF, nutritional assessment form; APACHE, Acute Physiology and Chronic Health Evaluation; SOFA, Sequential Organ Failure Assessment; SAPS, Simplified Acute Physiology Score; CCI, Charlson Comorbidity Index.

**Table 2:** Multivariable regression analysis of factors associated with nutritional delivery.

Variable	β (95% CI)	*p*-Value
Caloric delivery (% of target)		
VBF+ vs. Control	31.2 (17.6 to 44.8)	**<0.001**
NAF: Moderate vs. Normal–Mild	24.2 (1.55 to 46.8)	**0.037**
NAF: Severe vs. Normal–Mild	25.4 (1.86 to 49.0)	**0.035**
APACHE II (per point)	0.00 (−0.89 to 0.89)	1.000
CCI (per point)	0.00 (−1.62 to 1.62)	1.000
Protein delivery (g/kg/day)		
VBF+ vs. Control	0.168 (0.040 to 0.297)	**0.011**
NAF: Moderate vs. Normal–Mild	0.153 (−0.017 to 0.322)	0.077
NAF: Severe vs. Normal–Mild	0.136 (−0.074 to 0.346)	0.202
APACHE II (per point)	0.001 (−0.010 to 0.011)	0.870
CCI (per point)	−0.008 (−0.031 to 0.015)	0.495

For caloric delivery, estimates are median regression coefficients (quantile regression, τ = 0.5) with bootstrap 95% CI (1000 replications), given the non-normal patient-level distribution (Shapiro–Wilk *p* < 0.001, skewness = 0.98). For protein delivery, estimates are Ordinary Least Squares regression coefficients with 95% CI, given the approximately normal patient-level distribution (Shapiro–Wilk *p* = 0.414, skewness = 0.06). Both models were adjusted for nutritional assessment (NAF), APACHE II score, and Charlson Comorbidity Index. Bold values indicate statistical significance (*p* < 0.05). NAF, nutritional assessment form; APACHE, Acute Physiology and Chronic Health Evaluation; CCI, Charlson Comorbidity Index.

**Table 3:** Safety and complication outcomes.

Complication, n (%)	Intervention (n = 48)	Control (n = 48)	*p*-Value
Gastrointestinal complications			
Watery diarrhea (>5 times/day)	0 (0)	6 (12.50)	0.026
Diarrhea > 750 mL/day	0 (0)	5 (10.42)	0.056
Vomiting	0 (0)	5 (10.42)	0.056
Regurgitation	2 (4.17)	7 (14.58)	0.159
Aspiration	3 (6.25)	6 (12.50)	0.486
Gastric intolerance (GRV > 300 mL)	1 (2.08)	8 (16.67)	0.031
Refeeding syndrome/electrolyte imbalance			
Phosphate decrease of >30% or <0.6 mmol/L	6/12 (50.0)	4/10 (40.0)	0.691
Critical electrolyte abnormality	1 (2.08)	1 (2.08)	1.000

GRV, gastric residual volume. Critical electrolyte abnormality defined as magnesium < 0.75 mmol/L, phosphate < 0.8 mmol/L, or potassium < 3.5 mmol/L. Percentages for electrolyte outcomes were calculated among patients at risk for refeeding syndrome.

## Data Availability

The datasets generated and/or analyzed during the current study are not publicly available because they contain information that could compromise participant privacy and are subject to institutional ethics restrictions. De-identified data may be made available from the corresponding author upon reasonable request and with permission from the Institutional Review Board.

## Supplementary Materials

The following supporting information can be downloaded at https://www.mdpi.com/article/10.3390/medicina62081591/s1, Figure S1. Distribution of daily nutritional delivery at the observation level. Figure S2. Distribution of patient-level summary nutritional delivery. Figure S3. Distribution of propensity scores in the intervention and control groups. Figure S4. Love plots showing standardized mean differences before and after propensity score adjustment. Table S1. Volume-based catch-up feeding formula and caloric concentration escalation protocol (intervention phase only). Table S2. Daily energy delivery according to refeeding-risk category in the intervention and control groups: (A) percent of prescribed target; (B) absolute delivery (kcal/kg/day). Table S3. Daily protein delivery according to refeeding-risk category in the intervention and control groups. Table S4. Assessment of multicollinearity among variables included in the multivariable regression models. Table S5. Effect of the VBF+ protocol on nutritional delivery after propensity score-based adjustment.
