# Supplementary material for: A Risk-Stratified, Volume-Based Plus Enteral Nutrition Protocol with Semi-Elemental Formula in Critically Ill Surgical Patients: A Before-and-After Implementation Study
Source: Medicina (Kaunas). 2026 Aug 18;62(8):1591. doi: 10.3390/medicina62081591 (PMC13515000; doi:10.3390/medicina62081591)
Supplement: Supplementary file 1 [file medicina-62-01591-s001.zip › medicina-4486722-supplementary.pdf]

Supplementary Materials

A Risk-Stratified, Volume-Based Plus Enteral Nutrition Protocol with semi-elemental formula in Critically Ill Surgical Patients: A Before-and-After Implementation Study

Contents

Figure S1. Distribution of daily nutritional delivery at the observation level

Figure S2. Distribution of patient-level summary nutritional delivery

Figure S3. Distribution of propensity scores in the intervention and control groups

Figure S4. Love plots showing standardized mean differences before and after propensity score adjustment

Table S1. Volume-based catch-up feeding formula and caloric concentration escalation protocol (intervention phase only)

Table S2. Daily energy delivery according to refeeding-risk category in the intervention and control groups: (A) percent of prescribed target; (B) absolute delivery (kcal/kg/day)

Table S3. Daily protein delivery according to refeeding-risk category in the intervention and control groups

Table S4. Assessment of multicollinearity among variables included in the multivariable regression models

Table S5. Effect of the VBF+ protocol on nutritional delivery after propensity score–based adjustment

Supplementary Figures

Figure S1. Distribution of daily nutritional delivery at the observation level

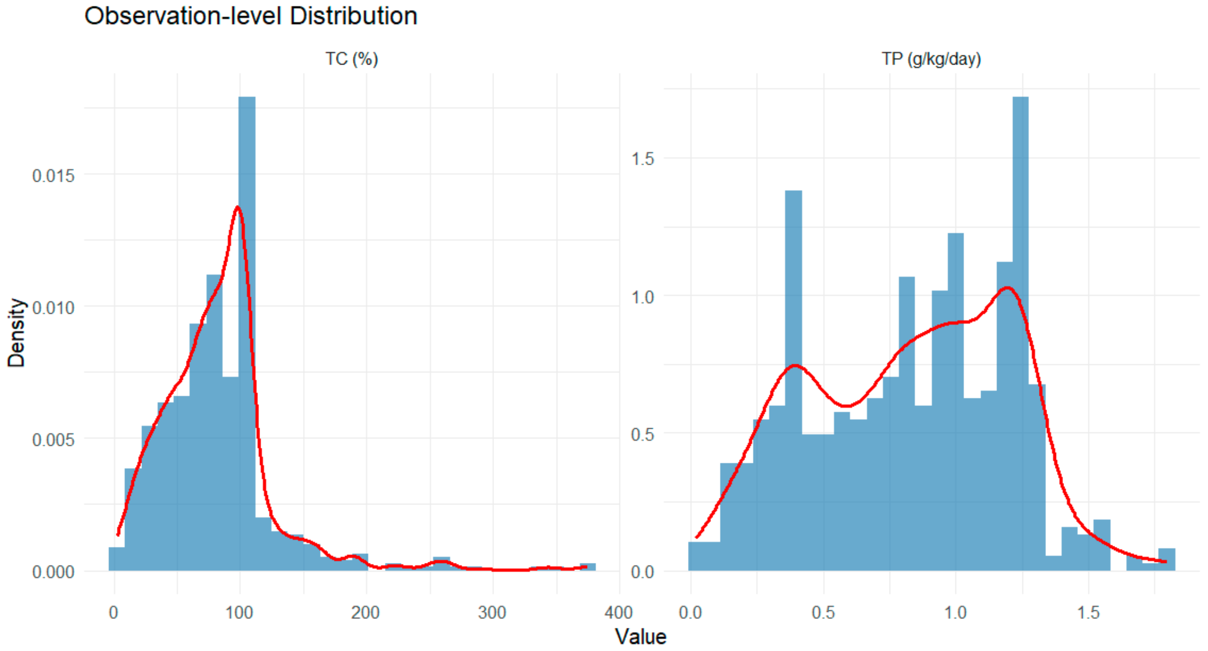

Histograms with overlaid kernel density curves showing the distribution of all daily nutritional delivery observations across the 10-day study period (n = 627 patient-days). TC(%): The left panel displays daily caloric delivery expressed as percentage of the prescribed caloric target. TP(g/kg/d): The right panel displays daily protein delivery expressed as grams per kilogram body weight per day (g/kg/day). Caloric delivery exhibited substantial right skewness (skewness = 2.11, Shapiro–Wilk p < 0.001), indicating a non-normal distribution. Protein delivery was approximately symmetric (skewness = −0.13).

Figure S2. Distribution of patient-level summary nutritional delivery

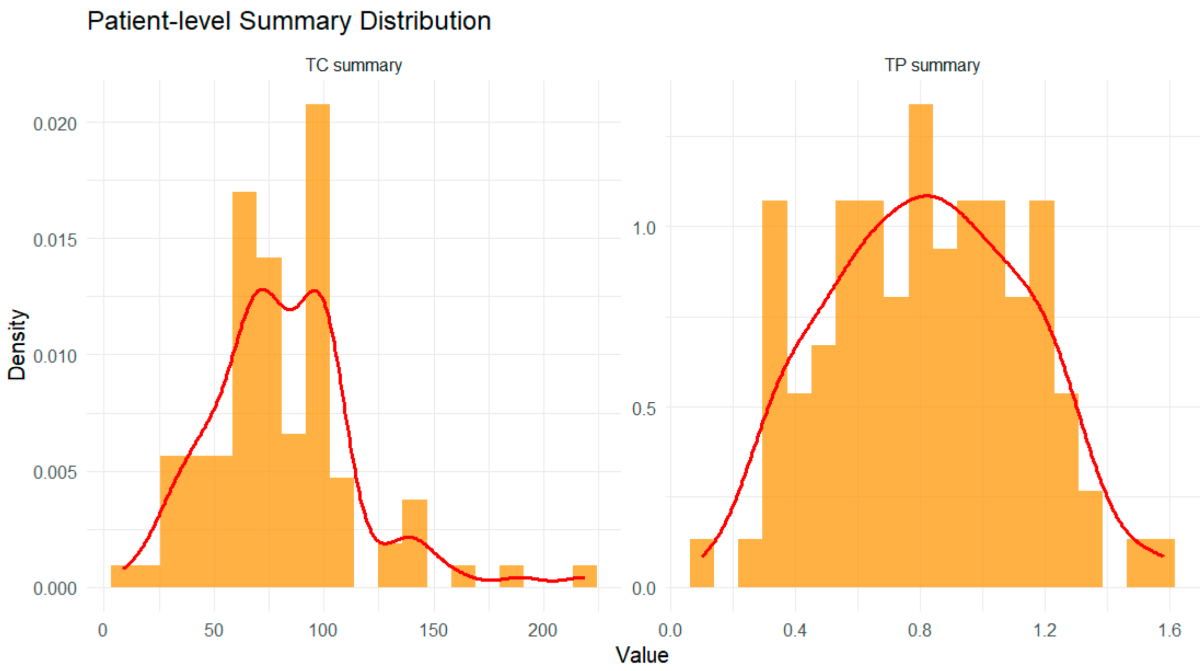

Histograms with overlaid kernel density curves showing the distribution of patient-level summary values used in the multivariable regression analysis (n = 96 patients). TC: The left panel displays the median of daily caloric delivery (% of target) per patient. TP: The right panel displays the mean of daily protein delivery (g/kg/day) per patient. The patient-level distribution of median caloric delivery remained non-normal (skewness = 0.98, Shapiro–Wilk p < 0.001), supporting the use of median regression (quantile regression at τ = 0.5) for the caloric delivery model. The patient-level distribution of mean protein delivery was approximately normal (skewness = 0.06, Shapiro–Wilk p = 0.414), supporting the use of multivariable linear regression for the protein delivery model.

Figure S3. Distribution of propensity scores in the intervention and control groups

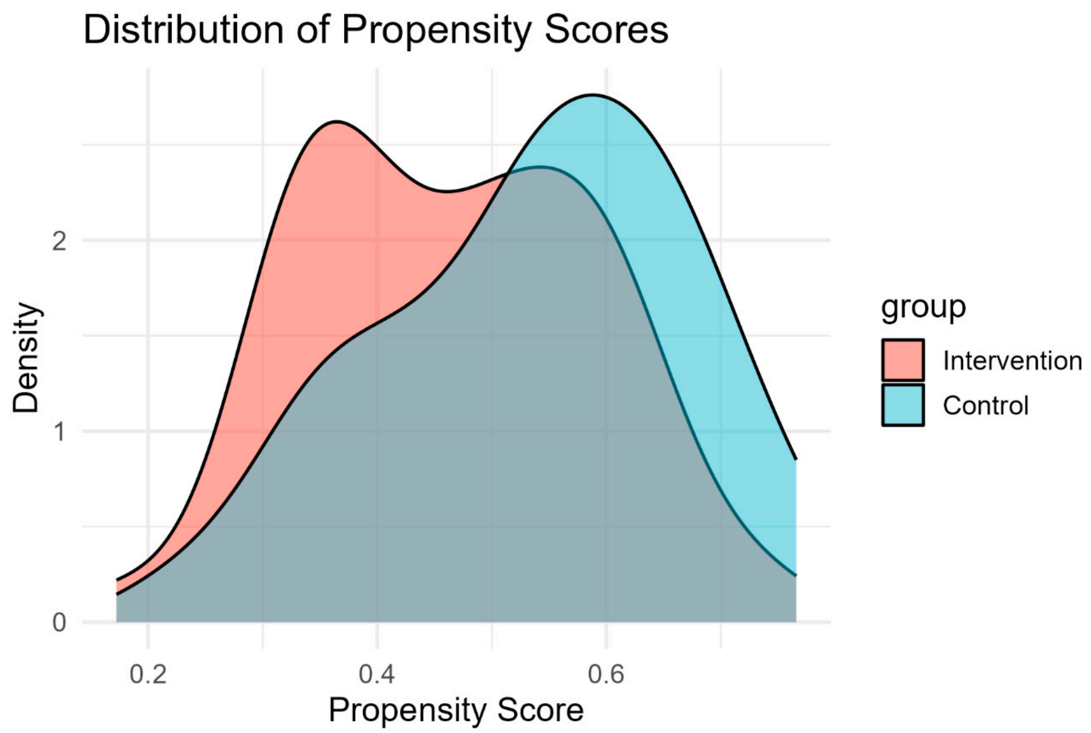

Density plots of estimated propensity scores in the intervention and control groups. Propensity scores were derived from a logistic regression model including age, sex, APACHE II score, Charlson Comorbidity Index, and refeeding risk category. The observed overlap between groups supports the positivity assumption required for propensity score analyses.

Figure S4. Love plots showing standardized mean differences before and after propensity score adjustment.

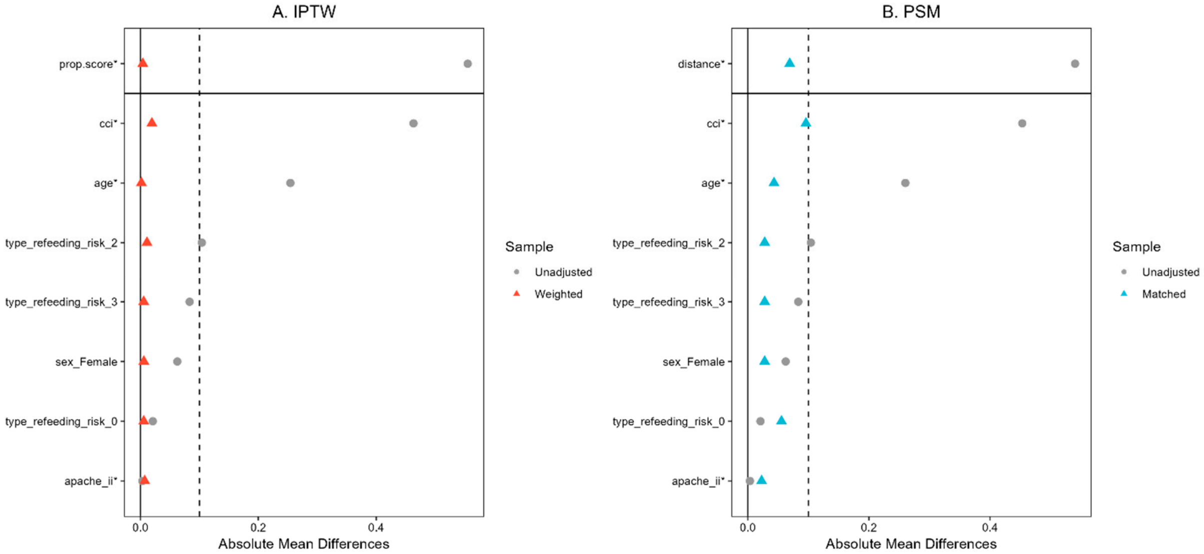

The left panel presents covariate balance before and after inverse probability of treatment weighting (IPTW), and the right panel presents balance before and after 1:1 nearest-neighbor propensity score matching (PSM). The dashed vertical line indicates the conventional threshold for acceptable covariate balance (absolute standardized mean difference = 0.10). After both IPTW and PSM, all baseline covariates achieved acceptable balance (absolute standardized mean differences < 0.10).

Supplementary Tables

Table S1. Volume-based catch-up feeding formula and caloric concentration escalation protocol (intervention phase only)

Formula concentration	Energy density	Triggering condition	Rationale
Standard	1.0 kcal/mL	Default; used for all patients at EN initiation	Starting concentration
Concentrated	1.5 kcal/mL	Compensatory rate >150 mL/h with standard formula	Achieves caloric target at lower infusion volume
High-density	2.0 kcal/mL	Compensatory rate >150 mL/h despite 1.5 kcal/mL formula	Maximizes caloric delivery within safe rate limits

Upon resumption of EN following any interruption, the bedside nurse recalculated the required infusion rate using the following formula: ***Compensatory Rate (mL/h) = Remaining Daily Volume (mL) ÷ Remaining Available Hours****, where the daily feeding cycle was defined as 06:00 to 06:00 of the following day. A maximum infusion rate of 150 mL/hour was enforced as a safety ceiling to minimize aspiration risk; if this threshold was exceeded, the ICU physician was notified to escalate the caloric concentration of the enteral formula according to the steps shown above. Concentration escalation was implemented at the ICU physician's discretion in conjunction with ongoing GRV monitoring. This protocol was applicable only in the intervention (VBF+) phase; in the control phase, infusion rates and formula concentration were adjusted at the treating physician's discretion without a standardized catch-up protocol.*

EN, enteral nutrition; GRV, gastric residual volume; VBF+, Volume-Based Plus protocol.

Table S2. Daily energy delivery according to refeeding-risk category in the intervention and control groups: (A) percent of prescribed target; (B) absolute delivery (kcal/kg/day)

(A)

Energy target (% of target)									
**Day**	**Intervention**					**Control**			p value
	**N**	No risk	Low risk	High risk	**N**	No risk	Low risk	High risk	
1	48	50.0 (47.1, 64.3)	92.3 (76.7, 125.5)	100.0 (100.0, 110.3)	48	40.6 (21.7, 85.0)	63.1 (43.3, 94.9)	124.4 (65.8, 210.6)	0.042
2	48	79.4 (66.7, 80.0)	100.0 (90.3, 100.8)	100.0 (100.0, 170.0)	48	54.9 (31.0, 66.6)	97.5 (47.4, 130.0)	230.6 (130.4, 302.4)	<0.001
3	48	83.3 (66.7, 84.8)	92.3 (78.7, 100.3)	100.0 (100.0, 142.6)	48	58.9 (33.6, 76.0)	96.0 (39.3, 109.8)	88.9 (62.1, 257.6)	<0.001
4	29	100.0 (92.8, 100.0)	92.8 (68.9, 99.2)	100.0 (98.0, 147.2)	43	64.1 (38.3, 77.4)	86.1 (43.1, 100.0)	94.8 (42.2, 188.5)	<0.001
5	23	100.0 (100.0, 100.0)	100.0 (88.9, 106.7)	98.0 (94.0, 100.6)	38	63.8 (44.1, 81.3)	74.3 (36.8, 104.2)	111.8 (14.3, 120.0)	<0.001
6	20	100.0 (100.0, 100.4)	98.9 (95.6, 103.8)	100.0 (100.0, 100.0)	36	70.7 (45.8, 78.8)	62.5 (25.6, 104.8)	44.4 (32.4, 104.9)	<0.001
7	18	100.0 (100.0, 100.0)	100.0 (98.9, 101.9)	100.0 (100.0, 100.0)	30	69.9 (50.4, 78.6)	62.5 (29.8, 70.6)	75.5 (56.6, 78.9)	<0.001
8	14	100.0 (100.0, 100.0)	100.0 (98.9, 101.9)	100.0 (100.0, 100.0)	24	71.4 (40.1, 81.5)	71.3 (59.8, 84.0)	65.2 (58.8, 81.7)	<0.001
9	13	100.0 (100.0, 100.0)	100.0 (87.0, 101.9)	100.0 (100.0, 100.0)	20	77.1 (64.3, 87.7)	80.2 (77.0, 88.2)	59.0 (39.8, 94.2)	<0.001
10	11	100.0 (100.0, 100.0)	101.9 (101.0, 102.9)	83.3 (75.0, 91.7)	20	82.9 (63.2, 90.7)	75.4 (68.4, 84.2)	81.5 (51.6, 119.0)	0.028
Total	48	80.0 (66.7, 100.0)	97.8 (85.3, 99.4)	100.0 (100.0, 127.9)	48	63.0 (44.2, 72.9)	77.5 (54.0, 96.6)	84.5 (57.9, 166.0)	<0.001

(B)

Energy target (kcal/kg/day)									
**Day**	**Intervention**					**Control**			p value
	**N**	No risk	Low risk	High risk	**N**	No risk	Low risk	High risk	
1	48	10.0 (9.4, 12.9)	13.8 (11.5, 15.0)	10.0 (10.0, 11.0)	48	8.7 (6.1, 14.0)	9.5 (6.5, 14.2)	12.4 (6.6, 21.1)	0.045
2	48	19.9 (16.7, 20.0)	20.0 (17.1, 20.2)	10.0 (10.0, 17.0)	48	12.3 (9.0, 17.7)	19.5 (9.5, 26.0)	23.1 (13.0, 30.2)	0.036
3	48	25.0 (20.0, 25.5)	23.1 (19.7, 25.1)	15.0 (15.0, 21.4)	48	17.5 (9.9, 20.0)	24.0 (9.8, 27.4)	13.3 (9.3, 38.6)	0.001
4	29	30.0 (27.9, 30.0)	27.8 (19.0, 29.8)	20.0 (19.6, 29.4)	43	19.2 (11.5, 23.2)	25.8 (12.9, 30.0)	28.4 (8.4, 37.7)	<0.001
5	23	30.0 (30.0, 30.0)	26.7 (26.7, 30.0)	24.5 (23.5, 25.1)	38	19.1 (13.2, 24.4)	22.3 (11.0, 31.2)	30.0 (3.6, 31.8)	0.001
6	20	30.0 (30.0, 30.1)	29.7 (28.7, 31.2)	30.0 (30.0, 30.0)	36	21.2 (13.7, 23.6)	18.8 (7.7, 31.4)	13.3 (9.7, 31.5)	<0.001
7	18	30.0 (30.0, 30.0)	30.0 (29.7, 30.6)	30.0 (30.0, 30.0)	30	21.0 (15.1, 23.6)	18.8 (8.9, 21.2)	22.6 (17.0, 23.7)	<0.001
8	14	30.0 (30.0, 30.0)	30.0 (29.7, 30.6)	30.0 (30.0, 30.0)	24	21.4 (12.0, 24.4)	21.4 (17.9, 25.2)	19.6 (17.6, 24.5)	<0.001
9	13	30.0 (30.0, 30.0)	30.0 (26.1, 30.6)	30.0 (30.0, 30.0)	20	23.1 (19.3, 26.3)	24.1 (23.1, 26.5)	17.7 (11.9, 28.2)	<0.001
10	11	30.0 (30.0, 30.0)	30.6 (30.3, 30.9)	25.0 (22.5, 27.5)	20	24.9 (18.9, 27.2)	22.6 (20.5, 25.3)	24.5 (15.5, 35.7)	0.028
Total	48	25.5	26.7	20.0	48	17.7	19.1	19.6	<0.001

		(17.8, 30.0)	(18.7, 30.0)	(11.0, 30.0)		(9.7, 23.1)	(9.6, 26.3)	(11.5, 33.5)	

Values are presented as median (interquartile range). Refeeding-risk categories were defined according to the study protocol as no risk, low risk, and high risk. The P values compare the intervention and control groups for each study day using the Wilcoxon rank-sum test, irrespective of refeeding-risk category. Table A reports caloric delivery as a percentage of the prescribed target, which by design is calculated against a denominator that varies by ICU day and refeeding-risk category (Figure 2); apparent values above 100% (and, in isolated instances, above 200%) reflect patients whose absolute intake exceeded a deliberately low early-phase or risk-adjusted target rather than a data error. Table B reports the corresponding absolute caloric delivery (kcal/kg/day) for the same patients, days, and risk categories, to allow the percent-of-target values in Table A to be interpreted alongside absolute intake.

Table S3. Daily protein delivery according to refeeding-risk category in the intervention and control groups

Protein target (g/kg/day)									
Day	Intervention					Control			p value
	N	No risk	Low risk	High risk	N	No risk	Low risk	High risk	
1	48	0.50 ± 0.26	0.64 ± 0.31	0.50 ± 0.34	48	0.44 ± 0.30	0.49 ± 0.39	0.62 ± 0.49	0.48
2	48	0.79 ± 0.26	0.80 ± 0.26	0.55 ± 0.20	48	0.55 ± 0.30	0.77 ± 0.43	0.88 ± 0.45	0.194
3	48	0.96 ± 0.19	0.88 ± 0.22	0.74 ± 0.20	48	0.67 ± 0.31	0.82 ± 0.48	0.89 ± 0.64	0.024
4	29	1.16 ± 0.14	1.04 ± 0.36	0.97 ± 0.23	43	0.74 ± 0.33	0.93 ± 0.44	0.92 ± 0.65	<0.001
5	23	1.19 ± 0.17	1.17 ± 0.16	0.90 ± 0.29	38	0.78 ± 0.35	0.86 ± 0.50	0.74 ± 0.60	<0.001
6	20	1.22 ± 0.08	1.23 ± 0.15	1.17 ± 0.10	36	0.83 ± 0.29	0.85 ± 0.61	0.74 ± 0.45	<0.001
7	18	1.22 ± 0.04	1.24 ± 0.04	1.22 ± 0.04	30	0.83 ± 0.28	0.66 ± 0.44	0.90 ± 0.40	<0.001
8	14	1.21 ± 0.03	1.24 ± 0.04	1.22 ± 0.02	24	0.82 ± 0.30	0.86 ± 0.14	0.83 ± 0.44	<0.001
9	13	1.21 ± 0.03	1.14 ± 0.20	1.23 ± 0.00	20	0.93 ± 0.25	1.07 ± 0.17	0.84 ± 0.45	<0.001
10	11	1.21 ± 0.03	1.25 ± 0.03	1.02 ± 0.29	20	0.99 ± 0.31	0.96 ± 0.23	1.05 ± 0.41	0.024
Total	48	0.86 ± 0.22	0.94 ± 0.20	0.75 ± 0.20	48	0.66 ± 0.25	0.77 ± 0.32	0.87 ± 0.40	0.017

Values are presented as mean ± standard deviation. Refeeding-risk categories were defined according to the study protocol as no risk, low risk, and high risk. The P values compare the intervention and control groups for each study day using Welch's two-sample t-test, irrespective of refeeding-risk category.

Table S4. Assessment of multicollinearity among variables included in the multivariable regression models

Variable	GVIF	df	GVIF^(1/(2×df))
**Outcome 1: Median caloric delivery (% of target) — median regression model**			
Group (Intervention vs. Control)	1.070	1	1.035
NAF (nutritional assessment)	1.183	2	1.043
APACHE II score	1.129	1	1.063
Charlson Comorbidity Index	1.120	1	1.058
**Outcome 2: Mean protein delivery (g/kg/day) — linear regression model**			
Group (Intervention vs. Control)	1.070	1	1.035
NAF (nutritional assessment)	1.183	2	1.043
APACHE II score	1.129	1	1.063
Charlson Comorbidity Index	1.120	1	1.058

All values refer to the generalized variance inflation factor (GVIF) and its adjusted form, GVIF^(1/(2×df)), which is equivalent to the square root of the standard VIF for binary predictors and is directly comparable across variables with different degrees of freedom. Values below 2.0 are considered acceptable.

GVIF, generalized variance inflation factor; df, degrees of freedom; NAF, nutritional assessment form; APACHE, Acute Physiology and Chronic Health Evaluation; CCI, Charlson Comorbidity Index.

Table S5. Effect of the VBF+ protocol on nutritional delivery after propensity score–based adjustment

Outcome	Analysis	β estimate	95% CI	p-value
Caloric delivery (% of target)	IPTW†	18.95	5.24 to 32.66	0.007
Caloric delivery (% of target)	PSM‡	29.30	18.50 to 41.00	0.001
Protein delivery (g/kg/day)	IPTW†	0.139	0. 028 to 0.250	0.015
Protein delivery (g/kg/day)	PSM‡	0.162	0.040 to 0.283	0.010

β estimates represent the adjusted between-group difference (intervention minus control); for caloric delivery, β represents the difference in the conditional median, whereas for protein delivery it represents the difference in the conditional mean; positive values indicate higher values in the intervention group. CI, confidence interval.
† IPTW: Estimates were obtained from survey-weighted linear regression using stabilized inverse probability of treatment weights estimated from the propensity score model. Effective sample sizes after weighting were 44.7 in the intervention group and 43.4 in the control group.
‡ PSM: Estimates were obtained from a 1:1 nearest-neighbor propensity score–matched cohort using a caliper width of 0.2 standard deviations of the logit of the propensity score. A total of 72 participants (36 matched pairs) were included in the matched analysis. Median regression (quantile regression at τ = 0.5) with 1000 bootstrap replications was used for caloric delivery, whereas linear regression was used for protein delivery.
Propensity scores were estimated using a logistic regression model including age, sex, APACHE II score, Charlson Comorbidity Index (CCI), and refeeding-risk category.
Abbreviations: IPTW, inverse probability of treatment weighting; PSM, propensity score matching; APACHE II, Acute Physiology and Chronic Health Evaluation II; CCI, Charlson Comorbidity Index; VBF+, Volume-Based Feeding Plus protocol.
